# Regularized least-squares migration of simultaneous-source seismic data with adaptive singular spectrum analysis

**DOI:** 10.1007/s12182-016-0134-1

**Published:** 2016-12-31

**Authors:** Chuang Li, Jian-Ping Huang, Zhen-Chun Li, Rong-Rong Wang

**Affiliations:** 1 School of Geosciences, China University of Petroleum, Qingdao, 266580 Shandong China; 2 Hisense (Shandong) Refrigerator Co. Ltd, Hisense, Qingdao, 266580 Shandong China

**Keywords:** Least-squares migration, Adaptive singular spectrum analysis, Regularization, Blended data

## Abstract

Simultaneous-source acquisition has been recognized as an economic and efficient acquisition method, but the direct imaging of the simultaneous-source data produces migration artifacts because of the interference of adjacent sources. To overcome this problem, we propose the regularized least-squares reverse time migration method (RLSRTM) using the singular spectrum analysis technique that imposes sparseness constraints on the inverted model. Additionally, the difference spectrum theory of singular values is presented so that RLSRTM can be implemented adaptively to eliminate the migration artifacts. With numerical tests on a flat layer model and a Marmousi model, we validate the superior imaging quality, efficiency and convergence of RLSRTM compared with LSRTM when dealing with simultaneous-source data, incomplete data and noisy data.

## Introduction

A fundamental factor considered in seismic data acquisition is efficiency. Simultaneous-source acquisition uses simultaneous shooting of two or more sources, resulting in the advantages of high efficiency and allowing denser source sampling and wider azimuths (Beasley 2008; Hampson et al. 2008). However, simultaneous shooting also produces blended data. There are mainly two ways to deal with simultaneous-source data. One is deblending the data (Mahdad et al. 2011; Chen et al. 2014; Chen 2015, 2016; Gan et al. 2016a; Zu et al. 2016) and then processing the deblended data with conventional methods. The other way is imaging the simultaneous-source data directly without separation (Tang and Biondi 2009; Berkhout et al. 2012; Chen et al. 2015). The velocity analysis of simultaneous-source data can also be implemented directly to obtain a precise velocity model in the common-midpoint domain (Gan et al. 2016b). The second approach has the advantage of high computational efficiency, but it suffers from migration artifacts because of the interference of adjacent sources.

Least-squares migration (LSM) is able to suppress the migration artifacts and produce high-quality images (Nemeth et al. 1999; Tang and Biondi 2009; Dai and Schuster 2013; Li et al. 2014, 2015a; Liu and Li 2015; Huang et al. 2013, 2015a). However, the computational cost of LSM is high as it is solved by gradient-based optimization schemes (Huang et al. 2015b; Huang and Zhou 2015; Li et al. 2016a). The computational efficiency and imaging quality can be improved by incorporating some sort of regularization into the LSM (Wang et al. 2009; Liu et al. 2013; Wang 2013; Li et al. 2015b; Lu et al. 2015). Structural constraint is an effective approach which can attenuate the migration artifacts while preserving the information of subsurface structures. Within angle-domain common-image gathers, Kuehl and Sacchi (2003) propose to use a smoothing operator along the ray parameter axis to suppress migration artifacts. This approach can also be implemented with structure-preserving constraints to improve the migration results (Wang and Sacchi 2009). The angle-domain common-image gathers need more computation and storage, so Xue et al. (2015) employ structure-enhancing filtering (Liu et al. 2010; Swindeman and Fomel 2015) as a shaping regularization operator for effectively removing noise. The structure-enhancing filter is also used as a preconditioning operator that updates the image only along prominent dips (Chen et al. 2015; Dutta and Schuster 2015), but the success of this approach significantly depends on the estimated dips.

Motivated by the excellent denoising performance of singular spectrum analysis (SSA) (Sacchi 2009; Oropeza and Sacchi 2010, 2011; Huang et al. 2014), we propose to incorporate a regularization term using SSA into least-squares reverse time migration (LSRTM) that eliminates migration artifacts caused by simultaneous-source data, incomplete data and noisy data. In order to make the SSA more efficient for large models, we divide large inverted images into several subsections by small spatial windows. Another problem of SSA is the difficulty to properly truncate singular values. The singular values are always selected manually by some criterion, for example, the number of linear events in the analysis window (Oropeza and Sacchi 2011). So we introduce the difference spectrum theory for adaptively determining the proper number of useful components.

In this paper, we first derive the forward modeling and migration operator of simultaneous-source data, and then present the theory of regularized least-squares reverse time migration (RLSRTM). The numerical tests on a flat layer model and a Marmousi model were carried out to compare RTM, LSRTM and RLSRTM when dealing with simultaneous-source data, incomplete data and noisy data. The numerical tests demonstrate the validity and superiority of the proposed method.

## Method

### Modeling and migration of simultaneous-source data

The forward modeling operator of simultaneous-source data is first derived according to the single shot forward modeling. The relation between the observed seismic data without blending and the reflectivity model can be expressed as

1 $$\left[ {\begin{array}{*{20}c} {{\mathbf{d}}_{1} } \\ {{\mathbf{d}}_{2} } \\ \vdots \\ {{\mathbf{d}}_{N} } \\ \end{array} } \right] = \left[ {\begin{array}{*{20}c} {{\mathbf{L}}_{1} } \\ {{\mathbf{L}}_{2} } \\ \vdots \\ {{\mathbf{L}}_{N} } \\ \end{array} } \right]{\mathbf{m}}$$

where ${\mathbf{d}}_{i}$ and ${\mathbf{L}}_{i}$ denote the observed data and forward modeling operator related to the *i*th shot; **m** denotes the reflectivity model. In LSRTM, the forward modeling operator is a linear operator with the Born approximation (Dai et al. 2012).

Two or more sources are excited simultaneously in the simultaneous-source acquisition. Assuming there are *n* super shots in a two-dimensional survey and each super shot consists of *k* sources, the blended seismic data can be expressed as

2 $$\left[ {\begin{array}{*{20}c} {{\mathbf{D}}_{1} } \\ {{\mathbf{D}}_{2} } \\ \vdots \\ {{\mathbf{D}}_{n} } \\ \end{array} } \right] = \;\left[ {\begin{array}{*{20}c} {\sum\limits_{i = 1}^{k} {{\mathbf{d}}_{\;1,i\;} } } \\ {\sum\limits_{i = 1}^{k} {{\mathbf{d}}_{\;2,i\;} } } \\ \vdots \\ {\sum\limits_{i = 1}^{k} {{\mathbf{d}}_{\;n,i\;} } } \\ \end{array} } \right] = \left[ {\begin{array}{*{20}c} {\sum\limits_{i = 1}^{k} {{\mathbf{L}}_{\;1,i\;} } } \\ {\sum\limits_{i = 1}^{k} {{\mathbf{L}}_{\;2,i\;} } } \\ \vdots \\ {\sum\limits_{i = 1}^{k} {{\mathbf{L}}_{\;n,i\;} } } \\ \end{array} } \right]{\mathbf{m}}$$

where ${\mathbf{D}}_{j}$ represents the *j*th super shot while ${\mathbf{L}}_{\;j,i\;}$ represents the demigration (forward modeling) operator corresponding to the *i*th source in the *j*th super shot.

The sources in the simultaneous-source acquisition can be generated either completely simultaneous or nearly simultaneous. The nearly simultaneous shooting method is distinguished from the completely simultaneous shooting method by a nonzero time-delay between adjacent sources. Introducing the time-shifting matrix into Eq. (2), we get the forwarding modeling operator of the nearly simultaneous-source shooting method,

3 $$\left[ {\begin{array}{*{20}c} {{\mathbf{D}}_{1} } \\ {{\mathbf{D}}_{2} } \\ \vdots \\ {{\mathbf{D}}_{n} } \\ \end{array} } \right] = \left[ {\begin{array}{*{20}c} {\sum\limits_{i = 1}^{k} {{\varvec{\uptau}}_{1,i} {\mathbf{d}}_{1,i} } } \\ {\sum\limits_{i = 1}^{k} {{\varvec{\uptau}}_{2,i} {\mathbf{d}}_{2,i} } } \\ \vdots \\ {\sum\limits_{i = 1}^{k} {{\varvec{\uptau}}_{n,i} {\mathbf{d}}_{n,i} } } \\ \end{array} } \right]{ = }\left[ {\begin{array}{*{20}c} {\sum\limits_{i = 1}^{k} {{\varvec{\uptau}}_{1,i} {\mathbf{L}}_{1,i} } } \\ {\sum\limits_{i = 1}^{k} {{\varvec{\uptau}}_{2,i} {\mathbf{L}}_{2,i} } } \\ \vdots \\ {\sum\limits_{i = 1}^{k} {{\varvec{\uptau}}_{n,i} {\mathbf{L}}_{n,i} } } \\ \end{array} } \right]{\mathbf{m}}$$

where ${\varvec{\uptau}}_{j,i}$ denotes the time-shifting matrix corresponding to the *i*th source in the *j*th super shot. Equations (3) and (2) become equivalent when ${\varvec{\uptau}}_{j,i}$ equals to a unit matrix, which represents the completely simultaneous shooting method.

Then, we rewrite the forward modeling of the simultaneous-source data with a simplified form,

4 $${\mathbf{D}} = {\mathbf{Sm}}$$

where **S** denotes the forward modeling operator of the simultaneous-source data.

The adjoint of the forward modeling operator can be written as,

5 $${\mathbf{S}}^{\text{T}} = \left[ {{\mathbf{S}}_{1}^{\text{T}} ,{\mathbf{S}}_{2}^{\text{T}} , \ldots ,{\mathbf{S}}_{n}^{\text{T}} } \right] = \left[ {\sum\limits_{i = 1}^{k} {{\mathbf{L}}_{1,i}^{\text{T}} {\varvec{\uptau}}_{1,i}^{\text{T}} } ,\sum\limits_{i = 1}^{k} {{\mathbf{L}}_{2,i}^{\text{T}} {\varvec{\uptau}}_{2,i}^{\text{T}} } , \ldots ,\sum\limits_{i = 1}^{k} {{\mathbf{L}}_{n,i}^{\text{T}} {\varvec{\uptau}}_{n,i}^{\text{T}} } } \right]$$

where the superscript T denotes the conjugate transpose operator.

So the RTM operator of the simultaneous-source data is

6 $$\begin{aligned} {\mathbf{m}}_{\text{mig}} & = \left[ {{\mathbf{S}}_{1}^{\text{T}} ,{\mathbf{S}}_{2}^{\text{T}} , \ldots ,{\mathbf{S}}_{n}^{\text{T}} } \right]\left[ {\begin{array}{*{20}c} {{\mathbf{D}}_{1} } \\ {{\mathbf{D}}_{2} } \\ \vdots \\ {{\mathbf{D}}_{n} } \\ \end{array} } \right] = \sum\limits_{l = 1}^{n} {{\mathbf{S}}_{l}^{\text{T}} {\mathbf{D}}_{l} } = \sum\limits_{l = 1}^{n} {\sum\limits_{i = 1}^{k} {\sum\limits_{j = 1}^{k} {{\mathbf{L}}_{l,i}^{\text{T}} {\varvec{\uptau}}_{l,i}^{\text{T}} } {\varvec{\uptau}}_{l,j}^{{}} {\mathbf{d}}_{l,j} } } \\ & = \sum\limits_{l = 1}^{n} {\sum\limits_{i = 1}^{k} {{\mathbf{L}}_{l,i}^{\text{T}} {\varvec{\uptau}}_{l,i}^{\text{T}} } {\varvec{\uptau}}_{l,i}^{{}} {\mathbf{d}}_{l,i} } + \sum\limits_{l = 1}^{n} {\sum\limits_{i \ne j}^{k} {\sum\limits_{j = 1}^{k} {{\mathbf{L}}_{l,i}^{\text{T}} {\varvec{\uptau}}_{l,i}^{\text{T}} } {\varvec{\uptau}}_{l,j}^{{}} {\mathbf{d}}_{l,j} } } \\ \end{aligned}$$

where ${\mathbf{m}}_{\text{mig}}$ denotes the migration result of the simultaneous-source data. The first term in Eq. (6) is the image of subsurface structures while the second term is the cross-term noise.

### RLSRTM using SSA

LSRTM can produce high-quality and high signal-to-noise ratio (SNR) images by iteratively updating the migration results close to the real reflectivity model. On the basis of the construction of the forward modeling and the migration operator of the simultaneous-source data, the misfit function of RLSRTM can be written as,

7 $$J ({\mathbf{m}} )= \frac{ 1}{ 2}\sum\limits_{i = 1}^{n} {\left\| {{\mathbf{S}}_{i} {\mathbf{m}} - {\mathbf{D}}_{i} } \right\|^{ 2} } + \frac{\lambda }{ 2}{\mathbf{R}}({\mathbf{m}})$$

where $\lambda$ denotes the regularization parameter which controls the tradeoff between the data term residual and the regularization term. The regularization parameter can be evaluated from the L-curve whose corner is used as a suitable regularization parameter (Rezghi and Hosseini 2009). However, this approach needs to compute the inverse problem several times to plot the L-curve, so it is too expensive to be practical for LSRTM. We propose that an a priori $\lambda$ is selected to keep the ratio of the data term gradient to the regularization term gradient $\upgamma$ a fixed value and ${0 < \gamma < 1}$. Since the data residual will decrease with an increase in iteration, the regularization parameter should be dynamic to prevent oversize regularization. ${\mathbf{R}}({\mathbf{m}})$ represents the regularizer that imposes constraints on the solution ${\mathbf{m}}$. These constraints are used to ensure that ${\mathbf{m}}$ should be sparse or the reflectors in ${\mathbf{m}}$ should be sharp. Here, we assume that ${\mathbf{R}}({\mathbf{m}}){ = }\left\| {{\mathbf{Wm}}} \right\|^{2}$ is the weighted reflectivity model while the weighting matrix ${\mathbf{W}}$ would preserve the interfaces of subsurface structures and eliminate the noise, then Eq. (7) can be rewritten as,

8 $$J ({\mathbf{m}} )= \frac{ 1}{ 2}\sum\limits_{i = 1}^{n} {\left\| {{\mathbf{S}}_{i} {\mathbf{m}} - {\mathbf{D}}_{i} } \right\|^{ 2} } + \frac{\lambda }{ 2}\left\| {{\mathbf{Wm}}} \right\|^{2}$$

In this paper, we define ${\mathbf{Wm}}$ as the SSA denoising of ${\mathbf{m}}$. Generally, the seismic signals have better coherence compared with the noise, so the noise in the migration results can be eliminated by SSA (Sacchi 2009; Oropeza 2010). The gradient to solve Eq. (8) is,

9 $$\frac{{\partial J({\mathbf{m}})}}{{\partial {\mathbf{m}}}} = \sum\limits_{i = 1}^{n} {{\mathbf{S}}_{i}^{\text{T}} \left( {{\mathbf{S}}_{i} {\mathbf{m}} - {\mathbf{D}}} \right) + \lambda {\mathbf{W}}^{\text{T}} {\mathbf{Wm}}}$$

Both RLSRTM and LSRTM are performed iteratively using the preconditioned conjugate-gradient algorithm (Nemeth et al. 1999). Two preconditioners, illumination compensation (Plessix and Mulder 2004; Li et al. 2016b) and high-pass filtering (Li et al. 2016b), are employed to improve the migration results.

### Adaptive SSA denoising

The basic assumption made by SSA can be summarized in a few words. If the seismic data consist of *a* complex events, the associated Hankel matrix of the data is a matrix of rank *a* (Hua 1992). When the data contain noise, the rank of the Hankel matrix will increase. So the denoising problem of seismic records can be attributed to the rank reduction issues of the Hankel matrix (Sacchi 2009; Oropeza 2010). SSA denoising can be implemented with the following steps (Sacchi 2009; Oropeza 2010). First, apply Fourier transform to the inverted image,

10 $$M(x,k) = \frac{1}{2\pi }\int\limits_{ - \infty }^{ + \infty } {m(x,z)e^{ - ikz} dz}$$

where $m(x,z)$ denotes the imaging results.

Denote $M_{k} = [M_{1} ,M_{2} ,M_{3} , \ldots ,M_{{N_{x} }} ]^{\text{T}}$ as a spatial vector of a given wavenumber *k* of the signal. The vector can be organized in a Hankel matrix,

11 $${\mathbf{M}} = \left( {\begin{array}{*{20}c} {\begin{array}{*{20}c} {M_{1} } \\ {M_{2} } \\ \vdots \\ {M_{{L_{x} }} } \\ \end{array} } & {\begin{array}{*{20}c} {M_{2} } \\ {M_{3} } \\ \vdots \\ {M_{{L_{x} + 1}} } \\ \end{array} } & {\begin{array}{*{20}c} \cdots \\ \cdots \\ \ddots \\ \cdots \\ \end{array} } & {\begin{array}{*{20}c} {M_{{K_{x} }} } \\ {M_{{K_{x} + 1}} } \\ \vdots \\ {M_{{N_{x} }} } \\ \end{array} } \\ \end{array} } \right)$$

where $N_{x}$ represents the number of traces of the imaging results, and $L_{x}$ and $K_{x}$ are selected to make the Hankel matrix approximately square. Here, $L_{x} = N_{x} /2 + 1$, $K_{x} = N_{x} - L_{x} + 1$.

Then, apply singular value decomposition (SVD) to the Hankel matrix,

12 $${\mathbf{M}} = {\mathbf{U}}{\varvec{\upsigma}}{\mathbf{V}}_{{}}^{\text{T}}$$

where ${\varvec{\upsigma}}$, ${\mathbf{U}}$, ${\mathbf{V}}$ denotes the singular values matrix and singular vectors associated with the Hankel matrix.

A key problem of SSA is the difficulty to properly truncate singular values. In this paper, we introduce the difference spectrum theory which can effectively reflect the difference of singular values of the useful components and noise. Assuming the diagonal components of the singular values matrix are denoted by $(\sigma_{1} ,\sigma_{2} ,\sigma_{3} , \ldots ,\sigma_{j} )$, the difference spectrum of singular values is defined as,

13 $$\begin{aligned} &{\mathbf{B}} = (b_{1} ,b_{2} , \ldots ,b_{j - 1} ) \hfill \\ &b_{i} = \sigma_{i} - \sigma_{i + 1} ,\quad i = 1,2, \ldots ,j - 1 \hfill \\ \end{aligned}$$

The difference spectrum reflects the changes of two adjacent singular values, and the peak position in the difference spectrum refers to the abrupt change point of singular values. For a noise-free migration image containing *a* complex events, the associated Hankel matrix of the data is a matrix of rank *a*, and the peak of the difference spectrum will exist at the *a*th point. Compared with the useful signals, the noise always has worse coherence and even smaller amplitude, thus corresponds to smaller singular values. In this case, the peak of the difference spectrum could be an effective indicator to preserve effective signals while maximizing noise attenuation. The criterion is same as using the numbers of linear events to truncate singular values (Oropeza 2010), but we implement it adaptively without human intervention. However, the difference spectrum may exhibit more than one peak value when the events are curved or the inverted image is complex, because the singular value components of the useful signals are dispersed. In order to minimize this problem, adaptive SSA denoising must be applied using windows in space. In short windows, it is possible to consider a curved event as linear. And, if multiple peaks cannot be avoided, we will use the last peak point for the consideration of preserving effective signals. Some examples of the adaptive SSA denoising are shown in the next section to test its validity.

If the peak value of the difference spectrum is $b_{a}$, the first *a* largest singular values are intercepted to reconstruct the Hankel matrix,

14 $${\mathbf{M}}_{a} = {\mathbf{U}}_{a} {\varvec{\upsigma}}_{a} {\mathbf{V}}_{a}^{\text{T}}$$

Once the rank reduced Hankel matrix is obtained, the next step entails reconstructing the inverted image by averaging components of the Hankel matrix across its anti-diagonals (Sacchi 2009). Finally, we apply an inverse Fourier transfer to the denoised data.

We should emphasize that SSA denoising needs additional computation, but the computational cost of SSA denoising is negligible compared with the cost of LSRTM. Even for a large size Hankel matrix, such as three-dimensional cases, it has been proven that dividing the data into small cubes and adopting the randomized singular value decomposition (RSVD) to perform the SVD can significantly improve the computational efficiency (Rokhlin et al. 2009; Oropeza and Sacchi 2010, 2011). In this paper, we focus on LSRTM for two-dimensional cases, so SVD is used in the following simulations.

## Examples

### Flat layer model

**Fig. 1:**
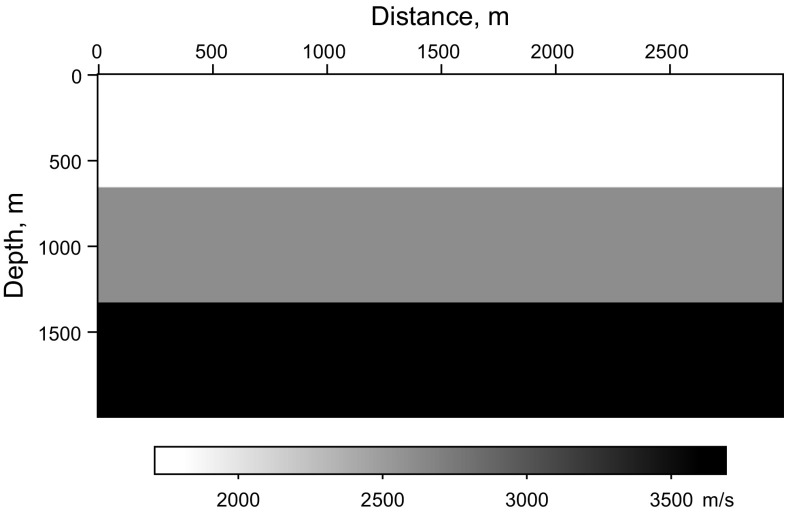
Velocity of the flat layer model

**Fig. 2:**
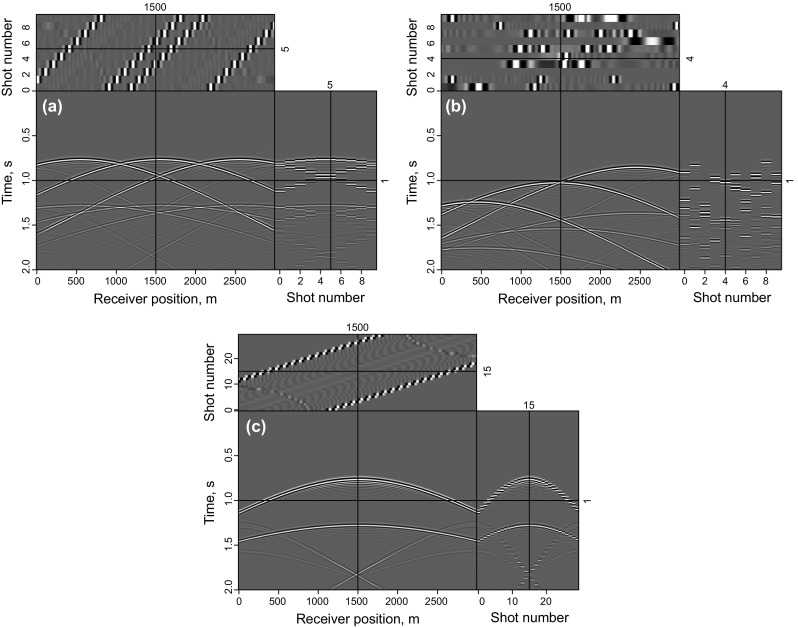
Synthetic data for the flat layer model: **a** simultaneous-source data with completely simultaneous shooting; **b** simultaneous-source data with nearly simultaneous shooting; and **c** common-shot data without blending

In this section, an imaging test of a flat layer model is implemented to demonstrate the validity of the proposed method and make a comparison between the completely simultaneous shooting method and the nearly simultaneous shooting method. In this example, 10 super shots are recorded by 300 receivers with a 10 m receiver interval. Each super shot contains three sources with a 100 m source interval. The real velocity shown in Fig. 1 is smoothed to be the migration velocity. The data simulated by the completely simultaneous shooting method and nearly simultaneous shooting method are shown in Fig. 2a, b. It can be seen that every super shot is blended with three single shots while there is a small time-delay between each single shot in Fig. 2b.

**Fig. 3:**
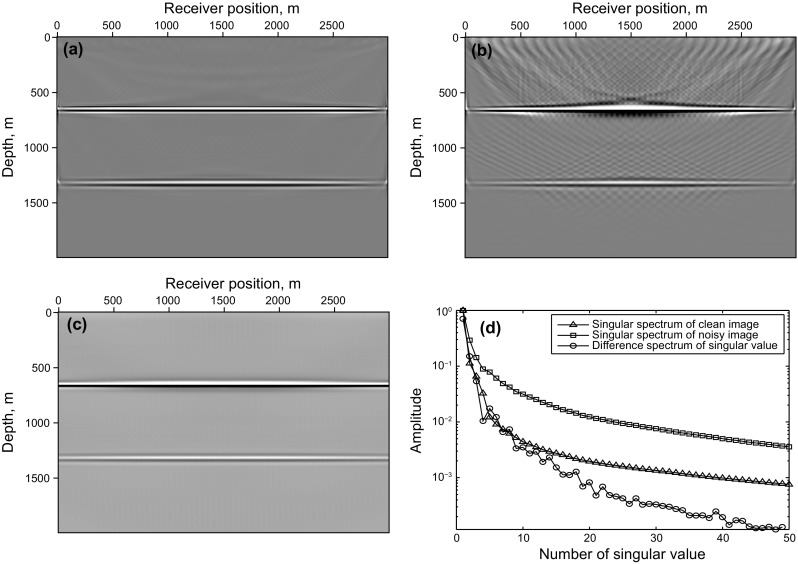
Synthetic test of adaptive SSA denoising with the flat layer model: **a** LSRTM result of the common-shot data in Fig. 2c; **b** RTM result of simultaneous-source data in Fig. 2a; **c** denoising result of **b**; **d** singular spectrum curves and its difference spectrum curve. Notice that the singular spectrum curves and the difference spectrum curves are plotted in semilogarithmic coordinates

Figure 3 shows the synthetic test of adaptive SSA denoising with the flat layer model. Figure 3a is a clean imaging result of the flat layer model, which is obtained by LSRTM of the common-shot data without blending (shown in Fig. 2c), Fig. 3b is the RTM result of simultaneous-source data (shown in Fig. 2a), and the denoising result of Fig. 3b is shown in Fig. 3c. Figure 3d shows the singular spectrum curves and the difference spectrum curve, which are plotted in semilogarithmic coordinates. All the singular spectrum curves in this paper are normalized by the first singular value. The useful signals mainly distribute in the first singular value component and the peak of the difference spectrum also exits in the first point. So the first singular value and its corresponding singular vector are used to recover the denoised data. It can be seen that the noise is effectively eliminated by adaptive SSA denoising.

**Fig. 4:**
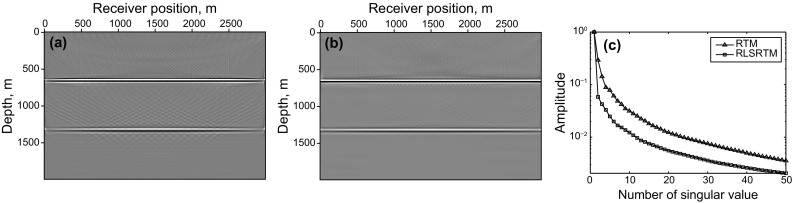
Imaging results of completely simultaneous-source data: **a** LSRTM result with 40 iterations; **b** RLSRTM result with 25 iterations; **c** singular spectrum curves from RTM result and RLSRTM result

**Fig. 5:**
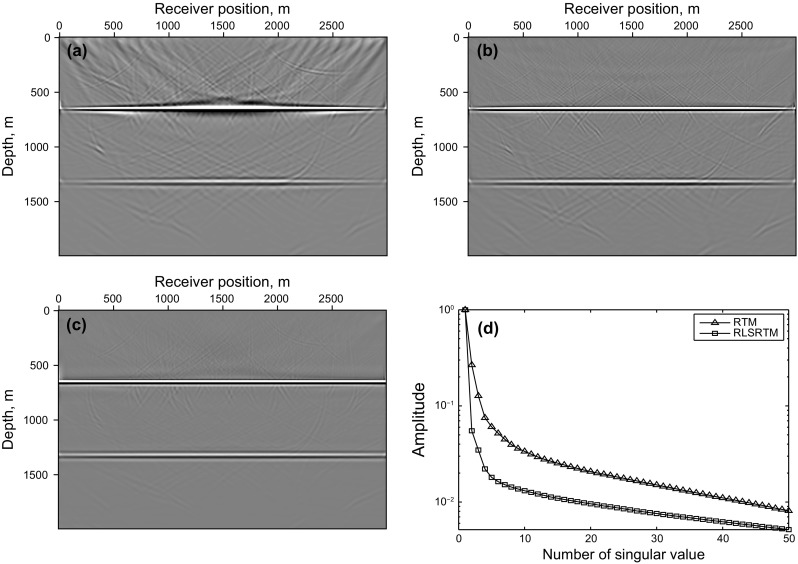
Imaging results of nearly simultaneous-source data: **a** RTM result; **b** LSRTM result with 40 iterations; **c** RLSRTM result with 25 iterations; **d** singular spectrum curves from RTM result and RLSRTM result

The imaging results of completely simultaneous-source data are shown in Fig. 4. Figure 4a shows the LSRTM result with 40 iterations which still suffers from some migration artifacts. Figure 4b shows the RLSRTM image with 25 iterations which exhibits higher quality image with less noise. The singular spectrum curves are plotted in Fig. 4c. The singular spectrum curve of RLSRTM result is more focused on the first point compared with RTM result, indicating that the noise is less. Figure 5 shows the imaging results of nearly simultaneous-source data in which we see similar results compared with Fig. 4. But the cross-term artifacts in Fig. 5 are a little weaker than those in Fig. 4, because stacking the migration results from different super shots can suppress the cross-term artifacts more effectively when the time-delay between adjacent sources is not zero. During the tests, the computer CPU was an Intel(R) Xeon(R) E5-2650 v2 @ 2.60 GHz and the running time of the serial program for LSRTM and RLSRTM with one iteration is 555 and 559 s, respectively.

Figures 4 and 5 demonstrate that (1) direct imaging of simultaneous-source data will introduce migration artifacts which are related to the time-delay between adjacent sources; (2) LSRTM and RLSRTM can suppress the migration artifacts and compensate for unbalanced illumination in the RTM image, but RLSRTM produces better images more efficiently compared with LSRTM.

### Marmousi model

**Fig. 6:**
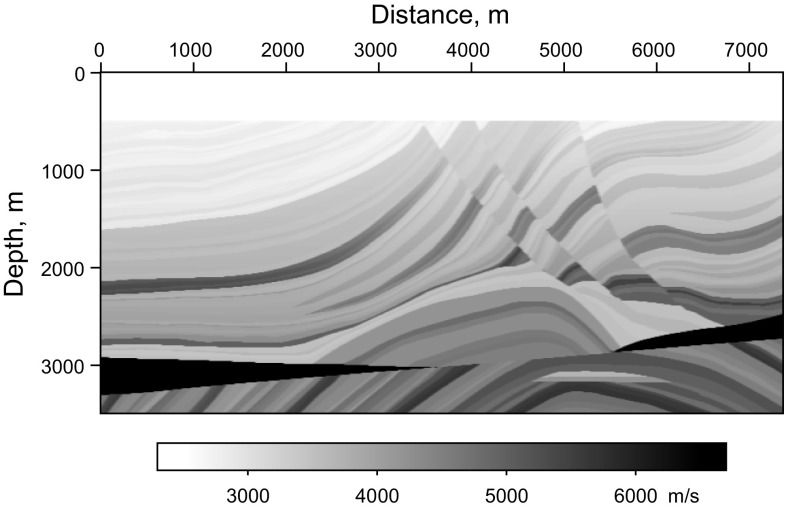
Velocity of Marmousi model

**Fig. 7:**
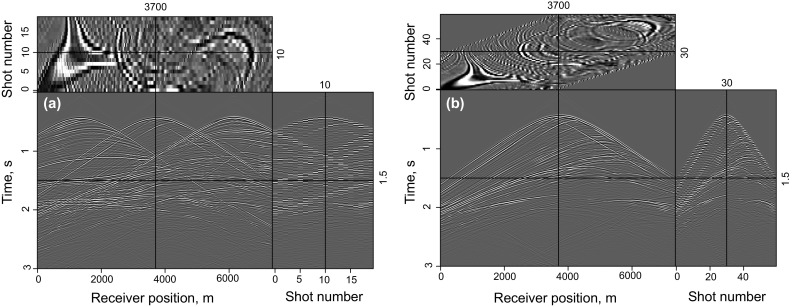
Synthetic data for the Marmousi model: **a** simultaneous-source data with completely simultaneous shooting method; **b** common-shot data without blending

We used a more realistic Marmousi model to test the proposed method (shown in Fig. 6). In this example, 20 super shots are simulated by firing three sources at the same time in each shot. The sources are distributed evenly with a 120 m source interval. The shot data shown in Fig. 7a are recorded by 737 receivers with a 10 m receiver interval. The real velocity shown in Fig. 6 is smoothed to be the migration velocity.

**Fig. 8:**
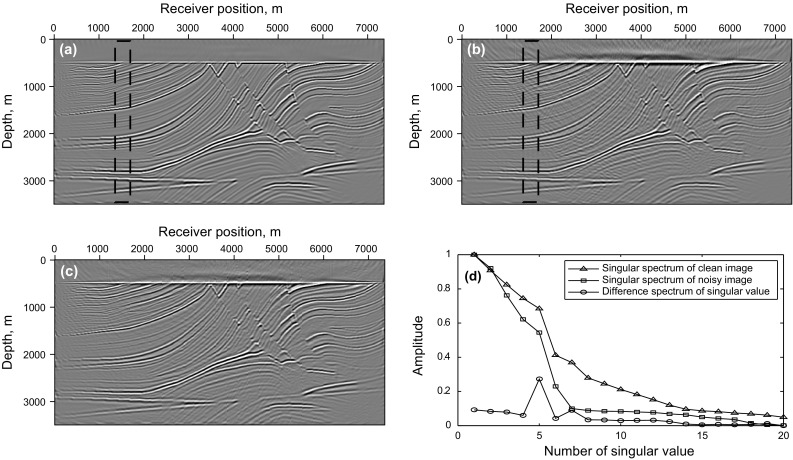
Synthetic test of adaptive SSA denoising with the Marmousi model: **a** LSRTM result of the common-shot data in Fig. 7b; **b** RTM result of the simultaneous-source data in Fig. 7a; **c** denoising result of **b**; **d** singular spectrum curves and the difference spectrum curve from the imaging results marked in the *black rectangle area*

We first test adaptive SSA denoising with the synthetic data of the Marmousi model, and the result is shown in Fig. 8. Figure 8a is the LSRTM result of the common-shot data without blending (shown in Fig. 7b), Fig. 8b is the RTM result of simultaneous-source data (shown in Fig. 7a), which contains obvious migration artifacts, and the SSA denoising result of Fig. 8b using spatial windows is shown in Fig. 8c. Thirty windows are selected to cover the entire image in space with 3500 m depth, overlapping every 10 traces. Figure 8d shows the singular spectrum curves and the difference spectrum curve from the imaging results marked by the black rectangle area. From the comparison of the singular spectrum of the clean image and the noisy image, it is clear that the useful signals mainly distribute in the first five singular value components while the noise mainly increases the scale of small singular value components. Thus, truncating the first five singular value components can preserve effective signals and suppress noise. As shown in Fig. 8c, most of the noise is suppressed after applying adaptive SSA denoising to each windowed image, but there is still some noise left on the image.

**Fig. 9:**
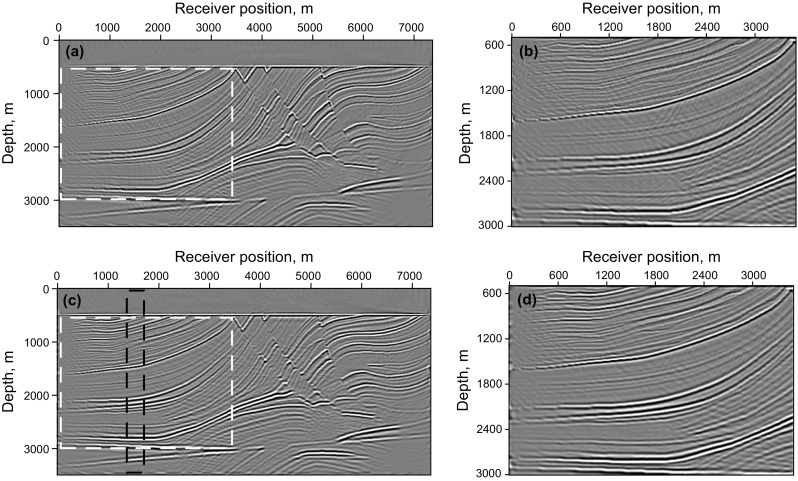
Imaging results of the simultaneous-source data: **a** LSRTM result with 40 iterations and **b** its zoom view; **c** RLSRTM result with 25 iterations and **d** its zoom view. The zoomed area is highlighted by the *white box*

**Fig. 10:**
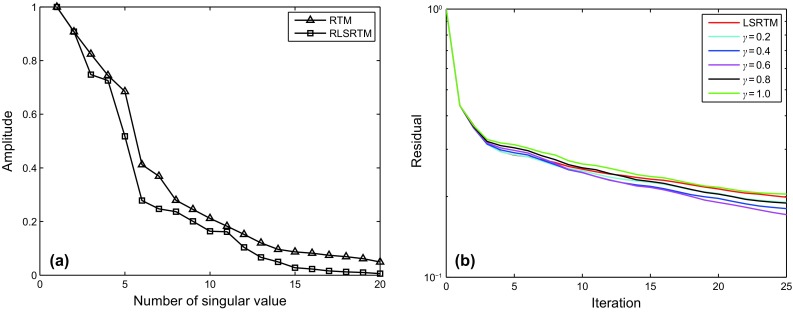
Singular spectrum curves and data residual convergence curves from the imaging results from simultaneous-source data: **a** singular spectrum curves from RTM and RLSRTM results; **b** data residual convergence curves with different regularization parameters. We only plot the singular spectrum curves from the imaging results marked by the *black rectangle* in Figs. 8a and 9c

An LSRTM image with 40 iterations and its zoom view are shown in Fig. 9a, b, which have higher imaging quality than RTM result, but still contain migration artifacts. Figure 9c, d shows the RLSRTM image with 25 iterations and its zoom view. The imaging quality of RLSRTM is comparable to LSRTM, but the noise in RLSRTM result is a little less. In the test, the running time of the serial program for LSRTM and RLSRTM with one iteration is 9094 and 9105 s, respectively. The singular spectrum curves from RTM and RLSRTM results marked by the black rectangle area in Figs. 8a and 9c are shown in Fig. 10a, in which the singular spectrum curves of RLSRTM result are closer to the singular spectrum curve of the clean image shown in Fig. 8d. In order to compare the convergence of LSRTM and RLSRTM, we present the data residual convergence curves for the simultaneous-source data with different regularization parameters in Fig. 10b. The convergence curves are plotted in semilogarithmic coordinates so that we can see the differences between LSRTM and RLSRTM more clearly. We notice that RLSRTM exhibits a faster convergence rate than LSRTM in the majority of cases. Only when $\upgamma\;{ = }\;\text{1}$ are the convergence rates of LSRTM and RLSRTM similar.

**Fig. 11:**
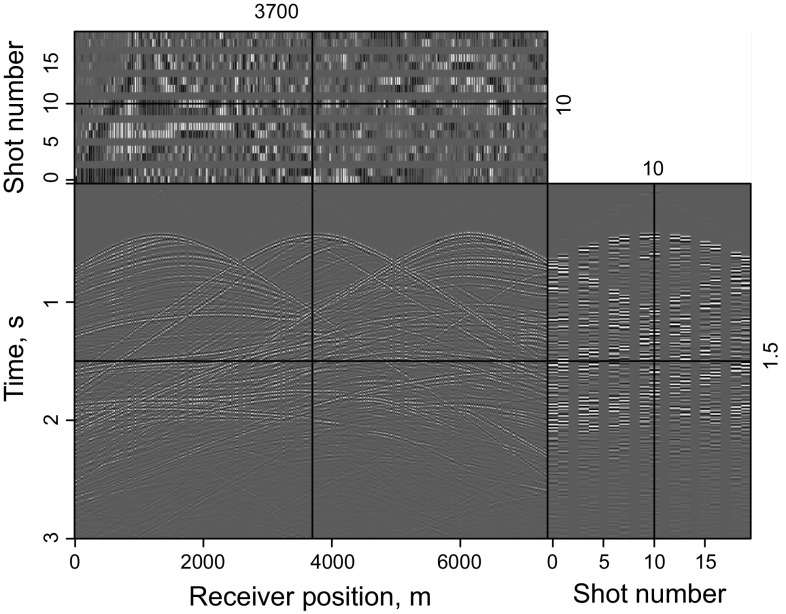
Synthetic data with 60% of the traces missing for the Marmousi model

**Fig. 12:**
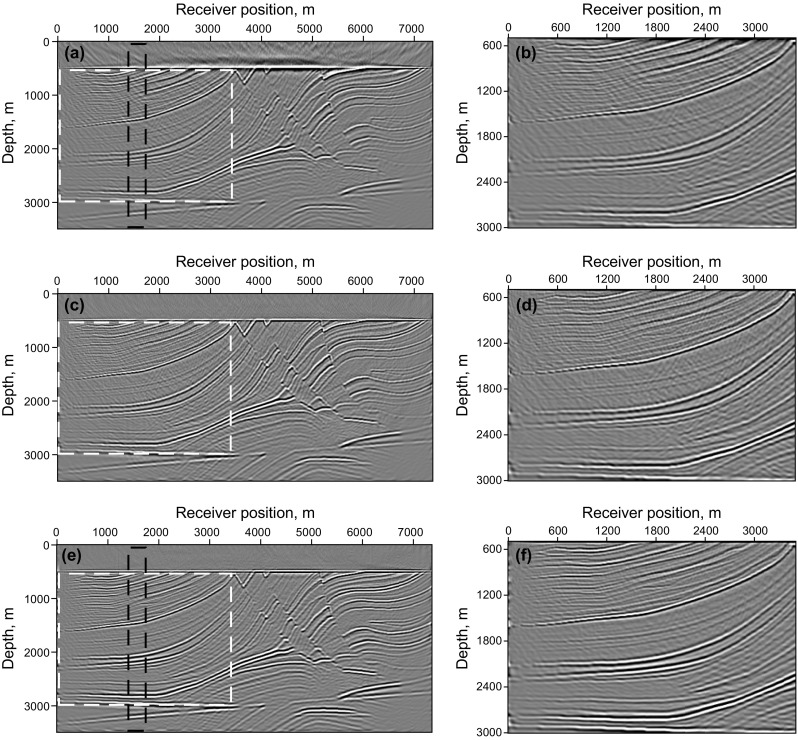
Imaging results of the incomplete data: **a** RTM result and **b** its zoom view; **c** LSRTM result with 40 iterations and **d** its zoom view; **e** RLSRTM result with 25 iterations and **f** its zoom view. The zoomed area is highlighted by the *white box*

**Fig. 13:**
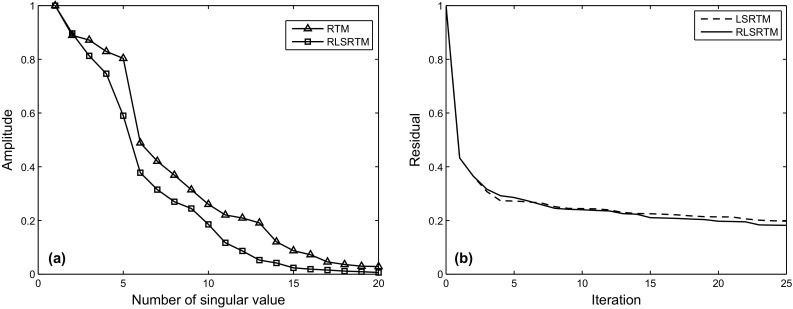
Singular spectrum curves and data residual convergence curves from the imaging results of the incomplete data: **a** singular spectrum curves from RTM and RLSRTM results; **b** data residual convergence curves. We only plot the singular spectrum curves from the imaging results marked by the *black rectangle* in Fig. 12a, e

Figure 11 shows synthetic data for the Marmousi model with 60% of the data missing. The RTM result of incomplete data shown in Fig. 12a, b contains more severe migration artifacts compared with the RTM result of the complete data. From the comparison of the results of LSRTM and RLSRTM in Fig. 12c–f, we draw the conclusion that LSRTM and RLSRTM can eliminate the migration artifacts caused by the incomplete data, while RLSRTM is more efficient in attenuating the migration artifacts compared with LSRTM. Figure 13a shows the singular spectrum curves from the RTM and RLSRTM results marked by the black rectangle area in Fig. 12a, e. The singular spectrum curve of RLSRTM result is more focused in the first few points than the singular spectrum curve of RTM result, indicating that RLSRTM result contains less noise. The data residual convergence curves for incomplete data are presented in Fig. 13b, which shows that both the data residuals of LSRTM and RLSRTM decrease fast and the convergence of RLSRTM goes a little faster.

**Fig. 14:**
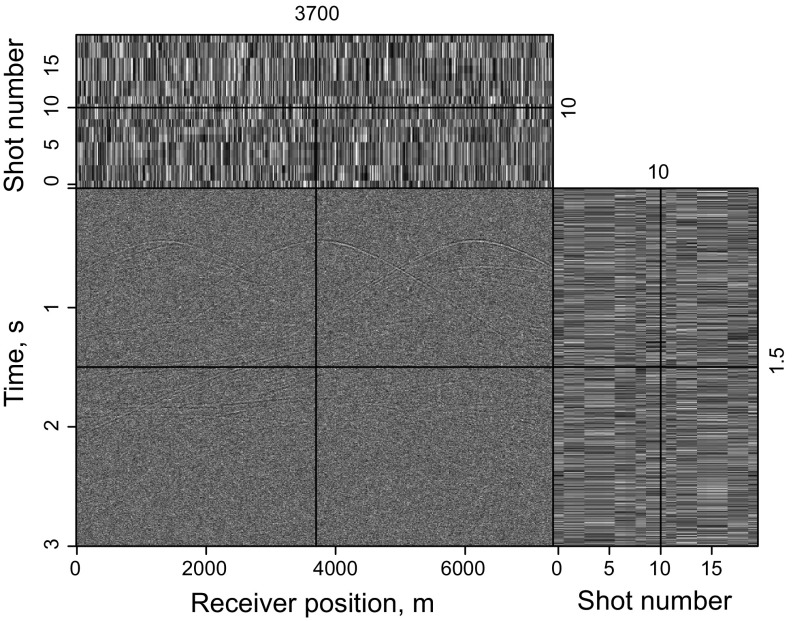
Synthetic data with random noise for the Marmousi model

**Fig. 15:**
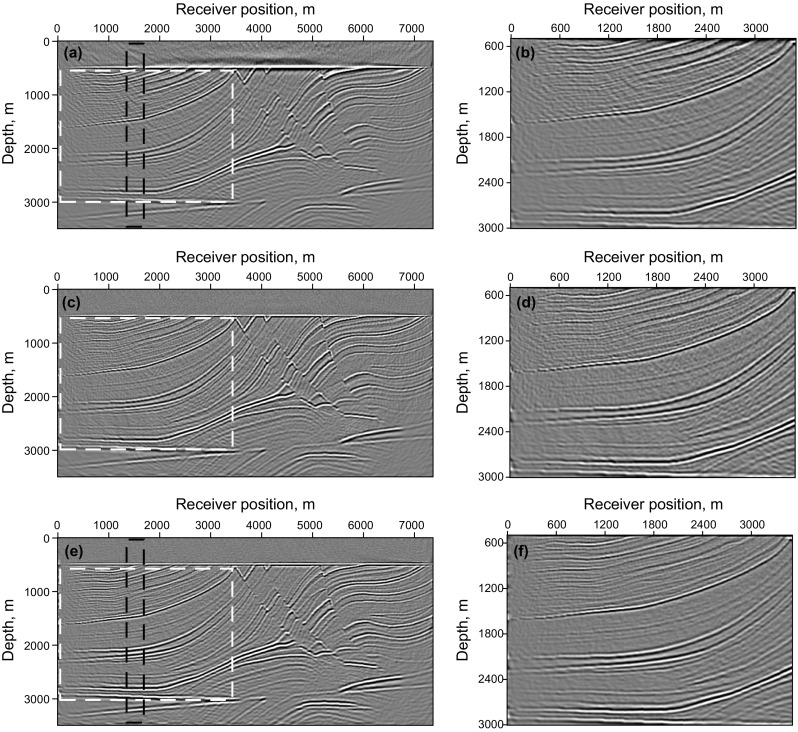
Imaging results of the noisy data: **a** RTM result and **b** its zoom view; **c** LSRTM result with 25 iterations and **d** its zoom view; **e** RLSRTM result with 25 iterations and **f** its zoom view. The zoomed area is highlighted by the *white box*

**Fig. 16:**
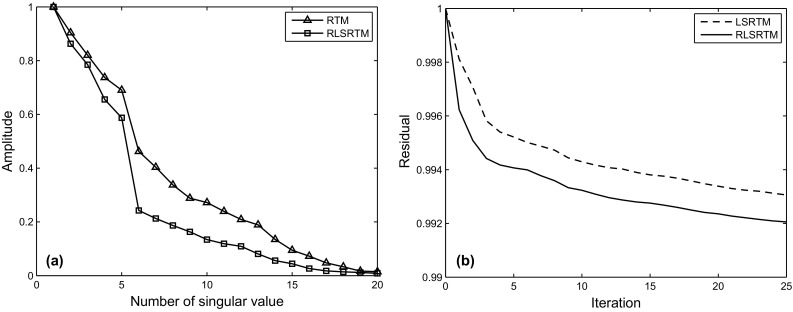
Singular spectrum curves and data residual convergence curves from the imaging results of noisy data: **a** singular spectrum curves of RTM and RLSRTM results; **b** data residual convergence curves. We only plot the singular spectrum curves from the imaging results marked by the *black rectangle* in Fig. 15a, e

Finally, an imaging test of noisy simultaneous-source data is presented. The noisy data in Fig. 14 are obtained by adding Gaussian noise into the simultaneous-source data with Eq. (15),

15 $$D_{\text{obs}} (x ,t )= D (x ,t )+ \delta \cdot {\text{rand}}({\text{size}}(D))$$

where ${\text{rand}}({\text{size}}(D))$ denotes the Gaussian noise, $\delta$ denotes the noise level. In this example, we want to test the robustness of the proposed method when the observed data contain strong noise, with $0. 1 { < }\delta { < 1} . 0$. The imaging results of noisy data are shown in Fig. 15. As shown in Fig. 15a, b, the Gaussian noise in the observed data also introduces slight random noise in the RTM images. The random noise cannot be suppressed but enhanced in the LSRTM image with 25 iterations, because the Gaussian noise cannot be predicted by the forward modeling operator, and will always remain in the data residual. However, the result of RSLRTM with 25 iterations in Fig. 15e, f exhibits less noise compared with the results of RTM and LSRTM. This demonstrates that RLSRTM is effective in producing high SNR images when the observed data suffer from severe Gaussian noise. Figure 16a shows the singular spectrum curves from the RTM and RLSRTM results marked by the black rectangle area in Fig. 15a, e. It is clear that the singular spectrum curve of RTM result is more dispersed because of the influence of the noise. Figure 16b shows the data residual convergence curves for noisy data. We find that the data residual of LSRTM and RLSRTM cannot be converged to below 0.9 because the severe noise will always remain in the data residual.

## Conclusions

We have proposed the regularized least-squares reverse time migration method using the adaptive SSA technique to solve the direct imaging problems of simultaneous-source data, incomplete data and noisy data. Difference spectrum theory is presented to implement SSA denoising adaptively. It is important to note that adaptive SSA denoising must be applied using spatial windows for better results when the underground structures are complex. The numerical tests on a flat layer model and a Marmousi model indicate that RLSRTM is able to eliminate migration artifacts efficiently and exhibits superior imaging quality and convergence compared with RTM and LSRTM.

This work can be easily extended to three-dimensional cases. We suggest that dividing the data into small cubes and adopting the RSVD (Rokhlin et al. 2009; Oropeza and Sacchi 2010, 2011) to perform the SVD could be useful to avoid the low computational efficiency problems of a huge Hankel matrix. In addition, the damped multichannel singular spectrum analysis (Huang et al. 2016) can attenuate more noise than traditional SSA. It can help improve the performance when there is random noise in the blended data. Our next work will take these methods into consideration.
